# A Retrospective Study on the Use of C-reactive Protein and Neutrophil-Lymphocyte Ratio as Predictive Markers to Prevent Unnecessary Appendicectomy

**DOI:** 10.7759/cureus.98921

**Published:** 2025-12-10

**Authors:** Harrison H Gregory, Baillie W Ferris, Michael C Auld

**Affiliations:** 1 Department of Surgery, Ipswich Hospital, Ipswich, AUS; 2 College of Medicine and Dentistry, James Cook University, Ipswich, AUS

**Keywords:** appendicectomy, appendicitis, biochemical markers, c-reactive protein, general surgery

## Abstract

Background: Symptomatology of acute appendicitis is a common presentation in children and biochemical markers are often utilised to differentiate acute appendicitis from other pathologies. The aim of this study is to assess the utility of C-reactive protein (CRP) and neutrophil-lymphocyte ratio compared to standard biochemical markers in predicting acute appendicitis and preventing unnecessary appendicectomy.

Methods: This is a single-centre retrospective cohort study of paediatric patients <16 years who presented to Ipswich Hospital with symptomatology of acute appendicitis between January 2019 to January 2024. Five hundred ninety-three patients were screened against inclusion/exclusion criteria and 582 patients were available for analysis.

Results: The study identified CRP (OR 1.009), neutrophil-lymphocyte ratio (NLR) (OR 0.909), neutrophil count (OR 1.222), and lymphocyte count (OR 0.708) to be significant coefficients in predicting acute appendicitis following logistic regression analysis. CRP was found to be a superior predictor of acute appendicitis following receiver operating characteristic (ROC) analysis (area under the curve (AUC) 0.712, sensitivity 75.60%, specificity 61.48%, p<0.001) compared to NLR, neutrophil and lymphocyte count.

Conclusion: In this large study, we have validated the effectiveness of certain biomarkers as predictive markers for acute appendicitis in children, potentially reducing the risk of unnecessary appendicectomy. CRP is superior to NLR, neutrophil and lymphocyte count in predicting acute appendicitis. The use of CRP, in addition to other markers such as NLR, is valuable in preventing unnecessary appendicectomy.

## Introduction

Acute appendicitis is a common indication for emergency abdominal surgery worldwide. The prevalence of acute appendicitis has increased by 20% between 1990 and 2019 [1]. The mainstay of treatment in Australia is appendicectomy, increasingly by laparoscopic approach. Australia has one of the highest rates of appendicectomy in the developed world - however, despite improvements in surgical technique, the complication rate associated with appendicectomy in the paediatric population is between 5-15% [2-4]. Negative appendicectomy rates for all ages approach 20% and have been shown to be associated with longer duration of hospital admission and higher costs to the healthcare system [5]. The rate of negative appendicectomy for the paediatric population in the Australian context has been scantily documented. Therefore, tools to identify which patients with symptomatology suggestive of appendicitis may not require surgical management are vital.

The paediatric population poses a unique diagnostic challenge to surgical clinicians. Paediatric patients are often poor historians and may have variable engagement with physical examination. Clinical assessment may also be confounded by understandable parental concerns [5,6]. These difficulties are further compounded by a reluctance to expose paediatric patients to radiation via computed tomography, as well as the operator-dependence and variability of ultrasonography in the diagnosis of appendicitis [6].

Strategies aimed at reducing the rate of negative appendicectomy have demonstrated a range of effectiveness in the literature. Several studies have explored the utility of biochemical markers in the diagnosis and risk stratification of acute appendicitis - certainly in the adult population, white cell (WCC) and neutrophil count, as well as C-reactive protein (CRP), have shown variable performance as a diagnostic tool [7-12]. More novel markers, such as neutrophil-lymphocyte ratio (NLR), have shown promise in predicting outcomes for other intra-abdominal pathologies such as acute diverticulitis [13]. The applicability of these biochemical markers is not well established in the paediatric population, despite this population being at a higher risk of negative appendicectomy and morbidity associated with operative intervention.

Given the lack of literature regarding biochemical marker use in the paediatric population, this study aims to identify the most effective biochemical marker in predicting acute appendicitis in paediatric patients presenting with symptomatology of acute appendicitis. The addition of a validated biochemical marker to the current diagnostic algorithm could more accurately demarcate paediatric patients requiring operative intervention to those not, reducing the risk of unnecessary appendicectomy in children and improving clinical outcomes. 

## Materials and methods

This is a single-centre retrospective cohort study of all patients aged between 0 and 16 years who underwent emergent appendicectomy at Ipswich Hospital, Queensland, between January 2019 to January 2024. This study was approved as a low/negligible risk study by the West Moreton Hospital and Health Service Human Research Ethics Committee following review. Inclusion criteria included all patients a) <16 years, b) who underwent emergent appendicectomy, and c) with baseline bloods performed at admission preoperatively. Exclusion criteria included patients with a) active malignancy or malignancy found on histopathology, b) recent use of exogenous steroids, granulocyte colony-stimulating factor, or cytotoxic chemotherapy, c) those who had received oral antibiotic therapy prior to admission, d) known immunodeficiency, or e) without bloods prior to operative intervention.

Demographic data including age, gender and Modified Monash Model (MMM) of their home address, and past medical history including the aforementioned comorbidities and medications, were collected from patients' integrated electronic health medical records. These variables were identified as potential confounders affecting biochemical markers at admission [14].

Independent continuous variables included WCC (x10^9^/L), neutrophil, lymphocyte and eosinophil count (x10^9^/L), NLR, neutrophil-eosinophil ratio (NER) and CRP level (mg/L). The dependent qualitative variable was post-operative histopathology demonstrating either acute appendicitis, or Enterobius vermicularis (E. vermicularis) or normal appendiceal tissue.

Data was analysed using Jamovi statistical software (The Jamovi Project, version 2.3). The Mann-Whitney U test was used to assess for significant differences in continuous variables between the two groups. Q-Q plots were used to identify non-normal distribution of continuous variables. One-way ANOVA test was used to identify significant differences in means between continuous variables with normal distribution, and Kruskal-Wallis test with Games-Howell post hoc analysis were used for continuous variables with non-normal distribution to identify significant differences. Logistic regression was then used to predict acute appendicitis on histopathology based on biochemical markers; covariates and confounders were excluded in a stepwise manner to identify significant regression coefficients.

Receiver operating characteristic (ROC) curve analysis was performed for CRP, NLR, neutrophil and lymphocyte count to assess their efficacy in predicting acute appendicitis on histopathology and thus need for surgical intervention. The Youden index was used to determine the optimal cut-off value for these diagnostic markers.

## Results

**Figure 1:**
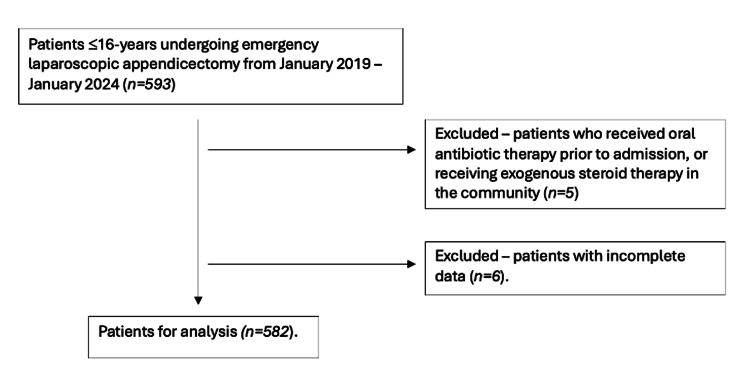
Flowchart of Cohort Selection.

The integrated electronic health medical records of 593 patients met the inclusion criteria and were screened accordingly. Eleven patients (1.85%) were removed from analysis due to meeting exclusion criteria or missing or incomplete data. In total, the data for 582 patients was analysed (Figure 1). 

The demographic, biochemical, and radiological details are described in Table 1 and organised according to histopathology, demonstrating either acute appendicitis or E. vermicularis or a normal appendix. Ultrasound results were collected by analysing the formal report completed by a specialist radiologist. All blood results were performed at a single laboratory and thus were standardised. Missing data was excluded via analysis by analysis on Jamovi. Generally, patients with appendicitis on histopathology demonstrated higher WCC and neutrophil counts, CRP and NLR value, whereas patients with normal appendiceal tissue or E. vermicularis demonstrated a higher eosinophil and lymphocyte count, and NER value.

**Table 1:** Demographics, baseline biochemical investigations and imaging findings for study population. MMM - Modified Monash Model, WCC - white cell count, NLR - neutrophil-lymphocyte ratio, NER - neutrophil-eosinophil ratio, CRP - C-reactive protein, USS - ultrasound

Characteristic/Variable		Normal Appendix / Enterobius vermicularis	Acute Appendicitis	P-value
Age (years)	Mean	11.43	11.85	0.145
	Median	12	12	
Gender	Male	56 (39.2%)	270 (61.5%)	<0.001
	Female	87 (60.8%)	169 (38.5%)	
MMM	Median	1	1	0.562
WCC (x10^9^)		9.67	12.47	<0.001
Neutrophils (x10^9^)		6.28	9.44	<0.001
Lymphocytes (x10^9^)		2.35	1.88	<0.001
Eosinophils (x10^9^)		0.26	0.17	<0.001
NLR		4.05	6.98	<0.001
NER		129.86	193.02	<0.001
CRP (mg/L)		17.27	44.75	<0.001
USS	Positive	16 (11.7%)	149 (34.7%)	0.089
	Negative	7 (5.1%)	16 (3.7%)	
	Equivocal	9 (6.6%)	6 (1.4%)	
	Not visualised	57 (41.6%)	71 (16.6%)	
	Not performed	48 (35.0%)	187 (43.6%)	

Mann-Whitney U test demonstrated significant differences in the WCC, neutrophil, lymphocyte and eosinophil count, NLR, NER, and CRP between patients demonstrating acute appendicitis on histopathology versus those with normal appendiceal tissue or E. vermicularis.

There were significant differences in all listed continuous variables between patients with appendicitis versus those with normal or E. vermicularis histopathology using Kruskal-Wallis testing and post-hoc Dwass-Steel-Critchlow-Fligner pairwise comparison (Tables 2, 3).

**Table 2:** Krusker-Wallis test for continuous variables (biochemical investigations). NLR - neutrophil-lymphocyte ratio, NER - neutrophil-eosinophil ratio, CRP - C-reactive protein, χ² - chi-square, df - degrees of freedom

Variable	χ²	df	P-value
Neutrophils (x10^9^)	48.0	1	<0.001
Lymphocytes (x10^9^)	24.4	1	<0.001
Eosinophils (x10^9^)	16.7	1	<0.001
NLR	49.5	1	<0.001
NER	16.2	1	<0.001
CRP (mg/L)	55.0	1	<0.001

**Table 3:** Post-hoc Dwass-Steel-Critchlow-Fligner pairwise comparison further reaffirmed significant differences in the above continuous variables between groups. NLR - neutrophil-lymphocyte ratio, NER - neutrophil-eosinophil ratio, CRP - C-reactive protein, W - Wilcoxon rank-sum test statistic

Variable	W	P-value
Neutrophils (x10^9^)	9.80	<0.001
Lymphocytes (x10^9^)	-6.98	<0.001
Eosinophils (x10^9^)	-5.77	<0.001
NLR	9.95	<0.001
NER	5.69	<0.001
CRP (mg/L)	10.5	<0.001

We identified a significant model for prediction of acute appendicitis on histopathology as shown in Table 4. Neutrophil count (OR 1.222, 95% CI 1.122-1.331, p <0.001), lymphocyte count (OR 0.708, 95% CI 0.538-0.931, p=0.013), NLR (OR 0.909, 95% CI 0.840-0.984, p=0.018), and CRP level (OR 1.009, 95% CI 1.002-1.015, p=0.01) were identified as significant predictors.

**Table 4:** Logistic regression model demonstrating coefficients for continuous variables in prediction of acute appendicitis on histopathology. NLR - neutrophil-lymphocyte ratio, NER - neutrophil-eosinophil ratio, CRP - C-reactive protein

Predictor	Coefficient Estimate	Standard Error	Z	P-value	Odds Ratio	95% Confidence Interval	
						Lower	Upper
Neutrophils (x10^9^)	0.20037	0.04366	4.589	<0.001	1.222	1.122	1.331
Lymphocytes (x10^9^)	-0.34593	0.13976	-2.475	0.013	0.708	0.538	0.931
Eosinophils (x10^9^)	-0.19144	0.40139	-0.477	0.633	0.826	0.376	1.814
NLR	-0.09551	0.04025	-2.373	0.018	0.909	0.840	0.984
NER	-3.05e−4	5.44e-4	-0.560	0.575	1.000	0.999	1.001
CRP (mg/L)	0.00873	0.00338	2.583	0.010	1.009	1.002	1.015

**Figure 2:**
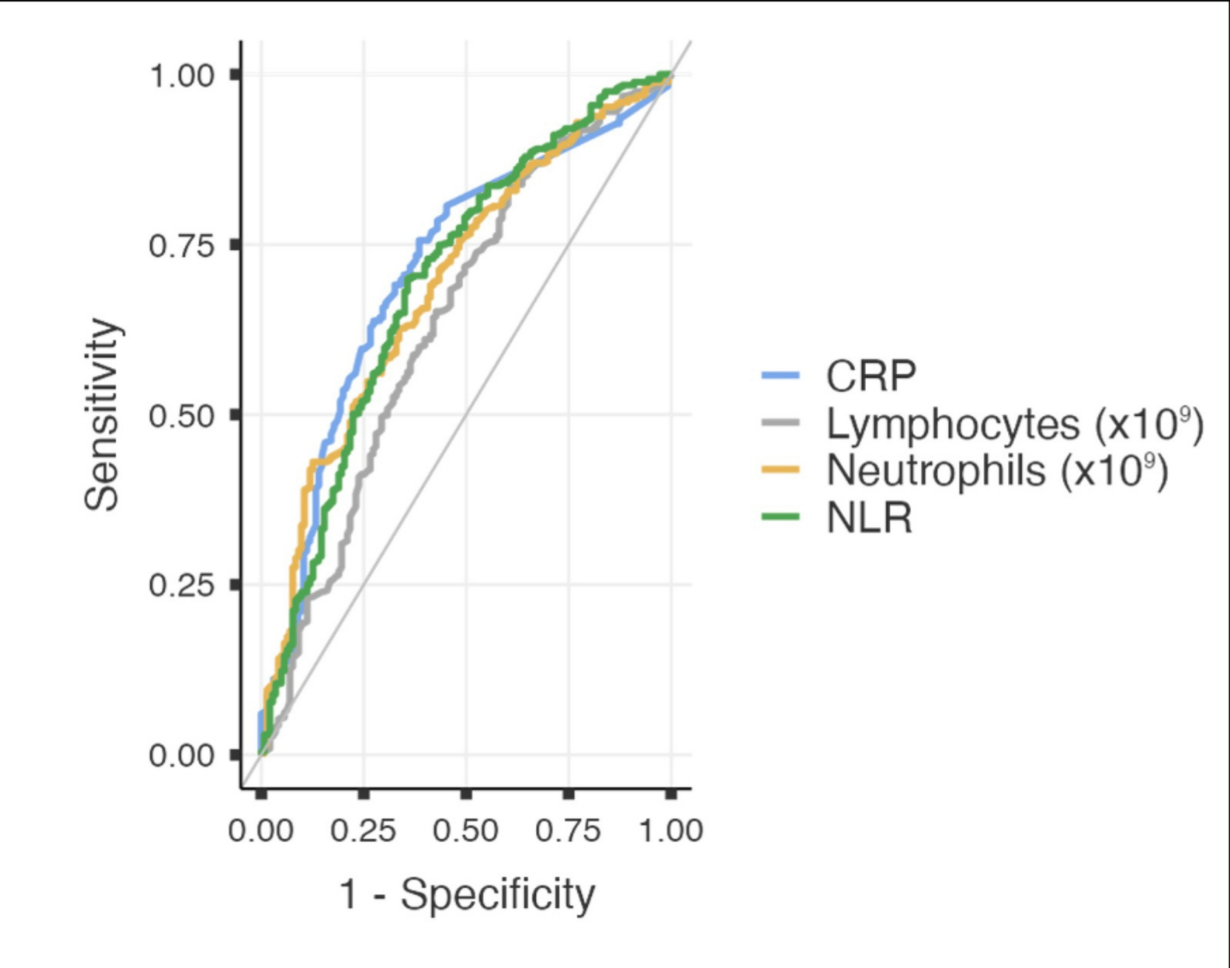
Receiver operating characteristic (ROC) analysis for significant predictive markers for acute appendicitis demonstrated on logistic regression model. CRP - C-reactive protein, NRL - neutrophil-lymphocyte ratio

ROC analysis showed CRP to be an effective diagnostic marker for prediction of acute appendicitis on histopathology, outperforming neutrophil and lymphocyte count and NLR (Figure 2, Table 5).

**Table 5:** Receiver operating characteristic (ROC) analysis for significant predictive markers for acute appendicitis demonstrated on logistic regression model. CRP - C-reactive protein, NLR - neutrophil-lymphocyte ratio, AUC - area under the curve, PPV - positive predictive value, NPV - negative predictive value

Predictor	Cut-Off	AUC	Sensitivity	Specificity	PPV	NPV	P-value
CRP (mg/L)	≥3.95	0.712	75.60%	61.48%	85.75%	45.11%	<0.001
NLR	≥2.87	0.696	69.93%	64.34%	85.75%	41.07%	<0.001
Neutrophils (x10^9^)	≥10.56	0.693	43.05%	80.84%	91.30%	33.33%	<0.001
Lymphocytes (x10^9^)	≤2.65	0.637	83.60%	39.16%	80.84%	43.74%	<0.001

## Discussion

Timely identification and delineation of paediatric patients presenting with symptomatology of acute appendicitis that require operative intervention is essential to limit unnecessary patient morbidity. This large single-centre retrospective cohort study aimed to assess the utility of biochemical markers such as CRP level and NLR on predicting post-operative histopathology, and by extension those patients who underwent unnecessary appendicectomy. This cohort of 582 patients represents the largest paediatric population evaluated within the current literature and Australian context. Our study supports the use of CRP as a predictive factor for acute appendicitis on post-operative histopathology, and by extension as a marker to delineate patients who do not require appendicectomy. A logistic regression model demonstrated CRP was significantly associated with the likelihood of acute appendicitis on histopathology (OR 1.009, 95% CI 1.002-1.015, p=0.001). A CRP cut-off of ≥3.95 demonstrated a sensitivity of 75.60% and specificity of 61.48% for prediction of acute appendicitis on histopathology (AUC 0.712, p<0.001). This model also supported the significance of neutrophil count (OR 1.222, 95% CI 1.122-1.331, p<0.001), lymphocyte count (OR 0.708, 95% CI 0.538-0.931, p=0.013) and NLR (OR 0.909, 95% CI 0.84-0.984, p=0.018), as similar predictors of these outcomes. These variables demonstrated reasonable sensitivity and specificity for predicting histopathology but failed to reach AUC >0.7. Following adjustment for known confounders (age, gender, MMM), there was no change to our findings from the regression model. Our study is the largest of its size for a paediatric population and is largely consistent with the existing body of literature.

The largest study prior to ours by Sarsu and Sarac largely supported our findings. Sarsu and Sarac [7] retrospectively assessed 417 patients aged between six and 17 years and categorised these patients into five groups: group 1 of healthy control patients; group 2 of patients with pain secondary to non-surgical pathology; group 3 of patients with normal histological appendices post-appendicectomy; group 4 of patients with histologically-confirmed acute appendicitis; and group 5 of patients with complicated appendicitis. They found statistically significant differences in WCC and CRP between groups 1-3 (8.8±2.6 and 0.4±0.7, 11.0±5.4 and 01.5±2.9, 13.3±5.7 and 3.0±4.5) and groups 4-5 (15.8±4.3 and 4.3±7.2, 16.5±4.5 and 9.7±10.1). Using a WCC cut-off of ≥13.3.1x109 and CRP cut-off of ≥6mg/L, Sarsu and Sarac found a sensitivity of 73.4% and 70.9%, and specificity of 80.0% and 68.7% respectively for diagnosing acute uncomplicated appendicitis (AUC WCC = 0.828, AUC CRP = 0.765) [7]. Msolli et al. [8] performed a similar retrospective study in which 500 patients of all ages (mean age = 28 years) were investigated. CRP level at admission and three hours post, as well as initial Modified Alvarado score (MAS), were identified, and their utility in predicting acute appendicitis on histopathology explored. Msolli et al. found grossly higher CRP values in patients with acute appendicitis compared to patients with normal appendices, but found poor performance of CRP with or without combination with MAS to predict histopathology following statistical analysis [8]. All ROC AUC, including CRP, and CRP plus MAS failed to exceed 0.7.

As opposed to our study, which did not identify WCC as a statistically significant predictor of patients with acute appendicitis, other authors have reported it as a useful marker in diagnosis. A systematic review and meta-analysis of earlier studies performed in 2013 by Yu et al. [9] examined seven studies with a total of 1011 patients of all ages and compared the diagnostic utility of procalcitonin, CRP and WCC in diagnosing acute appendicitis. Using a cut-off range for CRP between 30-60mg/L, and WCC 10-15.6x109, Yu et al. found a pooled sensitivity and specificity of 57% and 62% (AUC = 0.750), and 89% and 75% (AUC = 0.720) respectively [9]. A literature review of studies without ROC analysis by Shogilev et al. [10] in 2014 reported that a CRP cut-off of >10mg/L yielded a sensitivity between 65-85% and specificity between 59-73% in six studies [10]. 

Although our study found NLR a weaker predictor of histopathology, other authors demonstrated its potential utility in the diagnosis of acute appendicitis. Hajibandeh et al. [15] performed a systematic review and meta-analysis of seventeen studies including 8914 patients of all ages in 2020. Utilising a NLR cut-off value of 4.7, they produced a pooled sensitivity of 88.89% and specificity of 90.91%, with an AUC of 0.96 in diagnosing acute uncomplicated appendicitis [15]. With a cut-off of 8.8, NLR was 76.92% sensitive and 100% specific, with an AUC of 0.91 in diagnosing acute complicated appendicitis.

The results of our study, largely supported by the available literature, suggest CRP is an effective adjunct to assess the need for operative intervention in paediatric patients with clinical concern for appendicitis. The cut-off value of ≥3.95 provides a more specific marker to guide clinical decision-making in the paediatric population. CRP is an easily obtainable and cost-effective biochemical marker, and as such should be a routine investigation in children with clinical concerns for acute appendicitis. Likewise, NLR, although not as strong a marker as CRP, also shares these favourable characteristics, rendering it an attractive adjunct in the clinical algorithm. The strengths of this study are that the examined cohort represents the largest paediatric population in the current literature, as well as in the Australian context. Furthermore, our study compares the performance of a variety of commonly utilised markers including CRP and the less-used NLR, which is the current standard of care. These results suggest CRP and NLR can be reliably used as cost-effective biomarkers to avoid unnecessary surgical intervention in children. Despite these promising findings, it is important to acknowledge its limitations.

The retrospective design of this study increases the risk of selection bias. This selection bias is related to the exclusion of patients who did not undergo operative intervention. Given decision for operative intervention in paediatric patients remains largely driven by clinical acumen, the impact of biochemical markers preoperatively in patients with right lower quadrant pain may be under-valued. Another limitation lies in the inconsistency of biochemical marker cut-off values in the published literature - CRP as a marker alone had a documented cut-off of between >5 - >50 for establishing a diagnosis of acute appendicitis [7-11]; therefore, a standardised sensitivity and specificity for these biochemical markers is impractical for comparisons between studies. The large scale of this study and analytic methods utilised minimise these risks however, and as such the results are clinically significant.

A clear future direction for investigation would be whether a biochemical marker could be effective in predicting E. vermicularis infestation over a normal appendix in paediatric patients with lower abdominal pain. Although management of both E. vermicularis infection and non-surgical lower abdominal pain is non-operative, antihelminth therapy is indicated in patients with E. vermicularis. Obviously, timely provision of antihelminth therapy on discharge for these paediatric patients is optimal to improve patient outcomes.

Our study demonstrates CRP, and to a lesser extent NLR, as effective and affordable biomarkers in predicting acute appendicitis in paediatric patients, and by extension preventing the need for unnecessary appendicectomy. In conjunction with clinical acumen and radiological investigations, CRP can function to identify which paediatric patients can be appropriately managed non-operatively, preventing undue morbidity and improving clinical outcomes.

## Conclusions

This study aimed to determine the utility of various biochemical markers in predicting acute appendicitis in the paediatric population and by extension their effectiveness in reducing unnecessary appendicectomy. A CRP cut-off of ≥3.95 was effective in predicting acute appendicitis on histopathology. Other markers including neutrophil count, lymphocyte count and NLR demonstrated similar performance with respect to sensitivity and specificity, but failed to reach an AUC threshold of 0.7 and thus have limited clinical utility. Consideration of non-operative management in paediatric patients after specific consideration of CRP level, in conjunction with imaging and clinical acumen is warranted.
